# An In Vivo Predictive Dissolution Methodology (iPD Methodology) with a BCS Class IIb Drug Can Predict the In Vivo Bioequivalence Results: Etoricoxib Products

**DOI:** 10.3390/pharmaceutics13040507

**Published:** 2021-04-07

**Authors:** Isabel Gonzalez-Alvarez, Marival Bermejo, Yasuhiro Tsume, Alejandro Ruiz-Picazo, Marta Gonzalez-Alvarez, Bart Hens, Alfredo Garcia-Arieta, Greg E. Amidon, Gordon L. Amidon

**Affiliations:** 1 Department of Pharmaceutical Sciences, College of Pharmacy, University of Michigan, Ann Arbor, MI 48109, USA; isabel.gonzalez@umh.es (I.G.-A.); yasuhiro.tsume@merck.com (Y.T.); Bart.Hens@pfizer.com (B.H.); geamidon@umich.edu (G.E.A.); glamidon@med.umich.edu (G.L.A.); 2 Department Engineering Pharmacy Section, Miguel Hernandez University, San Juan de Alicante, 03550 Alicante, Spain; alejandro.ruizp@umh.es (A.R.-P.); marta.gonzalez@umh.es (M.G.-A.); 3 División de Farmacología y Evaluación Clínica, Departamento de Medicamentos de Uso Humano, Agencia Española de Medicamentos y Productos Sanitarios, 28022 Madrid, Spain; agarciaa@aemps.es

**Keywords:** gastrointestinal simulator, in vitro dissolution, BCS class II, weak base, dissolution modelling

## Abstract

The purpose of this study was to predict in vivo performance of three oral products of Etoricoxib (*Arcoxia*^®^ as reference and two generic formulations in development) by conducting in vivo predictive dissolution with GIS (Gastro Intestinal Simulator) and computational analysis. Those predictions were compared with the results from previous bioequivalence (BE) human studies. Product dissolution studies were performed using a computer-controlled multicompartmental dissolution device (GIS) equipped with three dissolution chambers, representing stomach, duodenum, and jejunum, with integrated transit times and secretion rates. The measured dissolved amounts were modelled in each compartment with a set of differential equations representing transit, dissolution, and precipitation processes. The observed drug concentration by in vitro dissolution studies were directly convoluted with permeability and disposition parameters from literature to generate the predicted plasma concentrations. The GIS was able to detect the dissolution differences among reference and generic formulations in the gastric chamber where the drug solubility is high (pH 2) while the USP 2 standard dissolution test at pH 2 did not show any difference. Therefore, the current study confirms the importance of multicompartmental dissolution testing for weak bases as observed for other case examples but also the impact of excipients on duodenal and jejunal in vivo behavior.

## 1. Introduction

After patent and/or exclusivity period expiry, generic products must demonstrate bioequivalence (BE) with the innovator product. Any further change on innovator or generic product needs the evaluation of the impact of those changes on product performance. In general, in vivo human BE studies are prescribed in regulatory guidelines unless a biowaiver approach (i.e., BE demonstration by applying an in vitro dissolution test) is claimed. This later situation is feasible for Biopharmaceutics Classification System (BCS) class 1 and 3 drug compounds or for BCS class 2 compounds with a validated level A in vitro in vivo correlation (IVIVC) [1,2]. In vivo predictive dissolution (iPD) methodologies have been widely recommended for achieving a IVIVC for these compounds that may be extremely influenced by the surrounding variables of the gastrointestinal (GI) tract [3,4,5].

Etoricoxib (ETO) is a weak base (pKa 4.96) exhibiting pH-dependent solubility with high solubility at pH < 3 [6,7]. Due to its low solubility in the pH 4–7 range and its high permeability, ETO is classified as a BCS class II compound. When dosed orally, etoricoxib is completely and rapidly absorbed, with an oral bioavailability up to 100% [8]. Oral absolute bioavailability was reported to be 83% [9] or practically complete [10] confirming its high permeability characteristics. Pharmacokinetics of ETO appear to be linear over the entire dose range (i.e., 30–240 mg [8]).

Weakly basic drugs with poor intrinsic solubility (BCS class II with basic property, BCS class IIb) quickly dissolve at gastric pH, but may precipitate upon entry in the upper small intestine due to the pH shift. Therefore, dissolved BCS class IIb drugs may be present in a supersaturated state but will have the tendency to precipitate when being transferred from the stomach to the small intestine.

In order to study the impact of (i) gastric emptying, (ii) secretion rates, and (iii) the physiological pH change in the GI tract on the drug dissolution profile, several iPD methods have been developed and the Gastro Intestinal Simulator (GIS) is one of the frequently used. The GIS structure includes three chambers, which aim to mimic the stomach, duodenum, and jejunum. Gastric contents, after formulation dosing, are pumped into the duodenal chamber with a pre-set gastric emptying half-life. Duodenal content is also pumped, into the jejunal chamber, at the adequate rate to keep its volume constant. The drastic pH changes triggered by gastric emptying in the fasted state (from pH 1−3 to the small intestinal pH 4−7) affect the dissolution and precipitation of weakly basic drugs. We have previously demonstrated with BCS class IIb drugs such as dipyridamole, dasatinib, and itraconazole [11,12,13] that he GIS model can adequately predict their in vivo dissolution and intestinal precipitation.

Our aim was to test three ETO products (i.e., one BE drug product, one non-BE product and the reference product) in the GIS to ascertain whether a more physiologically relevant dissolution method was able to reflect the in vivo BE outcome. In a final step, a computational model was used with the in vitro dissolution data to predict a plasma profile. In previous studies GIS dissolution profiles of BCS IIb compounds have been used coupled with PBPK models to forecast the plasma levels and the predictions have been compared with clinical published data, in this study for first time the formulations used in two Bioequivalence tests were used to reproduce in the GIS system the in vivo human outcome.

## 2. Materials and Methods

### 2.1. Chemicals

ETO (MW = 358.842 g/mol; logP = 2.79 and pKa: 4.96) [14] (ETO) active pharmaceutical ingredient (API) and the test and reference pharmaceutical products were kindly provided by a pharmaceutical company. All drug products were immediate release (IR) tablets containing 120 mg of ETO with conventional excipients in customary amounts. The reference product is commercialized in Europe as *Arcoxia^®^* and it contains calcium hydrogen phosphate (anhydrous) as excipient, which was not included in the candidate generic test products, which contained only microcrystalline cellulose as diluent. All other tablet core excipients (microcrystalline cellulose, magnesium stearate, and sodium croscarmellose) where qualitatively the same, but quantitatively different. Acetonitrile and methanol were purchased from Sigma (Barcelona, Spain). Sodium hydroxide (NaOH), sodium chloride (NaCl), and sodium dihydrogen phosphate monohydrate (NaH_2_PO_4_·H_2_O) were received from Sigma-Aldrich (St. Louis, MO, USA). Purified water (i.e., filtrated and deionized) was used in the analysis methods and in dissolution studies to prepare the dissolution media (Millipore, Billerica, MA, USA). 

### 2.2. In Vivo Studies

Data obtained from two BE cross-over studies in healthy subjects were available for comparison with in vitro data. The summary of the outcome of both studies is shown in Table 1. The study was approved by Ethic committee from Clinical Trial unit HUP (Hospital Universitario de la Princesa, Madrid, Spain; approval codes: XETO1x206 and XETO1x211)

Study 1 was a controlled, balanced, randomized, two-period crossover BE study using 48 healthy subjects. Study 2 was a single-blind, controlled, balanced, randomized, two-period crossover BE study including 36 healthy subjects. In each study, the volunteers received two products, one dose of the IR test product (120 mg) and one dose of the reference product (Arcoxia^®^ 120 mg) in a sequence determined by randomization. An adequate washout period was set between both arms in each study. Blood samples were taken for 72 h. ETO concentration in plasma samples was determined by a validated HPLC method in both studies. Plasma maximal concentration C_max_ and area under the curve, AUC_0–72h_ were calculated from the average or individual plasma concentration time profiles. AUC values were estimated individually by non-compartmental methods from the in vivo observations.

The product that failed to show BE is designated ETO NoBE in the manuscript and the one passing the BE standard limits is designated as ETO BE. Importantly, as the confidence intervals of C_max_ did not include the 100% value, the C_max_ of the generic products were statistically different from that of the reference product.

To allow the combination of data from different BE studies we used a normalization method based on the ratio of concentrations of reference products at each time point [15,16]. Similar normalization results were obtained by using the reference AUC ratios (data not shown).

### 2.3. Dissolution Experiments in GIS

**Figure 1:**
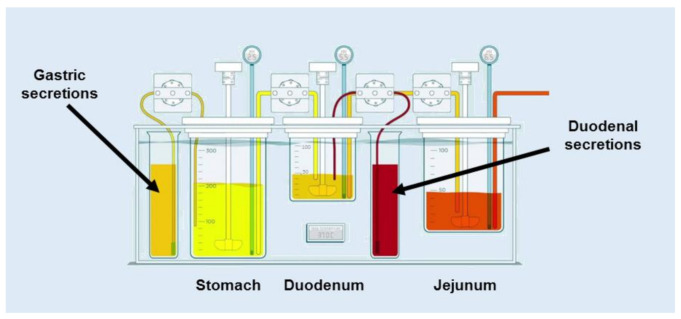
Setup and design of the GIS that was applied to test the different products of Etoricoxib, ETO in fasted state conditions. Adapted with permission of Reference [17]. Copyright 2018 Elsevier.

The GIS structure, containing a gastric chamber (GIS _Stomach_), (ii) a duodenal chamber (GIS _Duodenum_), and (iii) a jejunal chamber (GIS _Jejunum_) is represented in Figure 1. An additional computer control system is not represented.

A tablet of each ETO product was added to the stomach compartment at the start of the study. The dissolution media, initial volumes and secretion rates are described in Table 2.

Gastric emptying was set as a first-order kinetic process with a gastric half-life of 13 min, in accordance with the human gastric half-life reported values for liquids [18]. Duodenal volume was kept constant at 50 mL during the entire experiment. The jejunal compartment is empty at the start of the experiments and acts as the final accumulative receiver. As soon as the experiment was initiated, the fluid in the GIS_Stomach_ was transferred to the GIS_Duodenum_ via a transfer tube by a peristaltic pump (Ismatec REGLO pump; IDEX Health and Science, Glattbrugg, Switzerland). 

Four peristaltic pumps calibrated prior of the experiments are involved in GIS system. Two pumps introduce gastric and duodenal secretions in gastric and duodenal chambers respectively at a 1 mL/min flow. Duodenal content is kept constant by equilibration the output pumping rate with the input from stomach and the duodenal secretions. All the chambers are stirred with CM-1 overhead paddles (Muscle Corp., Osaka, Japan). In the duodenum and stomach stirring is set at a rate of 20 rotations per minute (RPM) alternating with a high-speed, quick burst every 25 s in order to mimic the contractions in stomach and duodenum. Jejunal chamber agitation was kept at a constant rate assuming weaker distal contractions of the intestinal tract. All the fluids were kept at 37 °C thanks to the thermostatic bath. Gastric volume was practically emptied at 60 min, thus all the pumps are shut down while the sampling procedure continues for up to 120 min. The pumps and overhead paddles were controlled by an in-house computer software program. Solution concentrations were determined by centrifuging 200 µL of the withdrawn sample for 5 min at a speed of 13,000× *g* (AccuSpin Micro 17, Fisher Scientific, Pittsburgh, PA, USA). After centrifugation, the supernatant was directly two-fold diluted with methanol to capture the dissolved fraction.

### 2.4. HPLC Analytical Method

ETO concentrations in samples were measured by HPLC-UV (Hewlett Packard series 1100 HPLC Pump combined with Agilent Technologies 1200 Series Autosampler and a ChemStation software (Agilent Technologies, B.04.03 version, Santa Clara, CA, USA). Injection volume was 50 µL and UV detection wavelength was 254 nm (Agilent 1100 Series UV lamp). Linearity was demonstrated between 50 µM and 500 µM with calibrated curves prepared in mobile phase from a ETO methanol stock solution (7 mM). Limit of detection (LOD) of and limit of quantification (LOQ) were 2.2 µM and 6.6 µM, respectively. The mobile phase was a mixture of 20:80 (*v*/*v*) water:acetonitrile both containing 0.1% (*v*/*v*) trifluoroacetic acid. After 2.3 min, the peak signal of ETO was observed using a reversed-phase C-18 column (Bondapak 250 mm × 4.6 mm—5 micron) and a 1 mL/min flow rate.

### 2.5. Analysis of the Mass Transport of ETO throughout the GIS 

The evolution of ETO concentrations in all chambers was described with a mass transport analysis approach (MTA) based on differential equations involving the dissolution, precipitation, and transit kinetics. The model was based on the previously described by Matsui and colleagues [19]. Slight modifications to account for ETO properties and its products (Table 3) were included. Equations are described in Supplementary materials.

The model was fit to the experimental data (ETO concentrations in all the chambers) using Phoenix WinNonlin V8 (Certara USA, Princeton, NJ, USA).

### 2.6. In Silico Simulations to Predict the Pharmacokinetic (PK) Profiles of ETO

**Figure 2:**
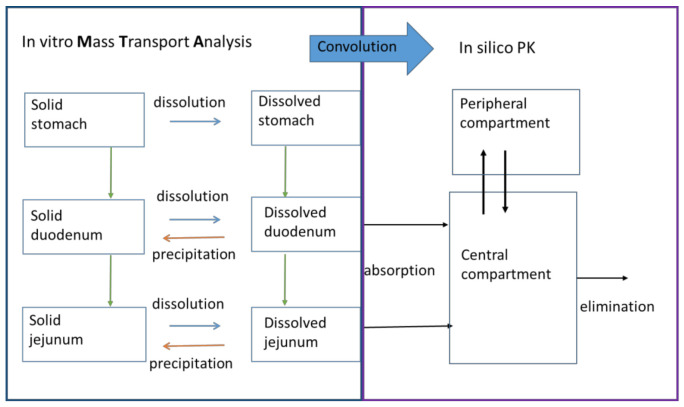
Mass transport analysis model (MTA) of the in vitro data as observed in the GIS with ETO products coupled with in silico pharmacokinetics (PK) parameters in order to simulate plasma profiles of ETO products.

A two-compartmental pharmacokinetic (PK) model was built in order to predict the plasma profiles after administration of ETO products. The design of the model is analog to the model as described by Matsui et al. with slight modifications to PK parameters (Figure 2). This model represents a central and peripheral compartment. Useful PK parameters to include in the model were extracted from intravenous (IV) data: 25 mg single-dose iv administration to six healthy volunteers [9] and 25 mg single-dose iv administration to 12 healthy volunteers [8], and oral data (120 mg tablets from [6]) to obtain indispensable values such as distribution/elimination rate constants in order to simulate distribution and elimination of ETO appropriately. The effective permeability (P_eff_) value in Caco-2 has been estimated in silico in two literature references using GastroPlus^TM^ software package (v. 9.7.0009, 2019, Simulations Plus Inc., Lancaster, CA, USA) as 4.75 × 10^−4^ cm/s [6] and 4.07 × 10^−4^ cm/s [7]. The highest estimation was used for the in vivo plasma level simulations. Simulated profiles were directly compared with the mean plasma profiles obtained from the in vivo bioequivalence studies. The PK parameters are listed in Table 4.

## 3. Results

### 3.1. Performance of the ETO Products in the GIS 

**Figure 3:**
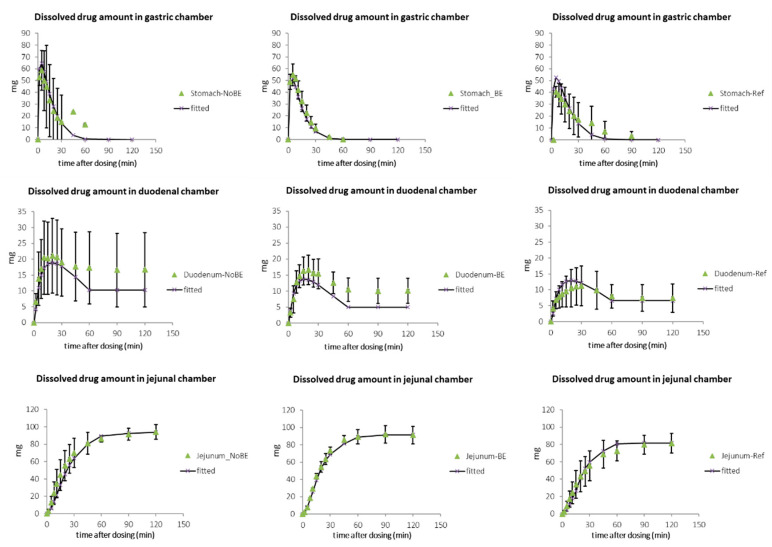
Experimental (green triangles) and model-predicted (grey symbols and line) values of amount dissolved (%) for Reference and Test BE and NoBE ETO formulations in each GIS chamber.

The average dissolved amounts of ETO in the three GIS chambers (1st–3rd row) for the three studied products (1st–3rd column) are represented in Figure 3 (solid lines correspond to the fitted values to the mass transport model). Each point corresponds to the average of four tablets of each formulation.

### 3.2. In Silico PK Model to Forecast the Systemic Performance of Oral Products

**Figure 4:**
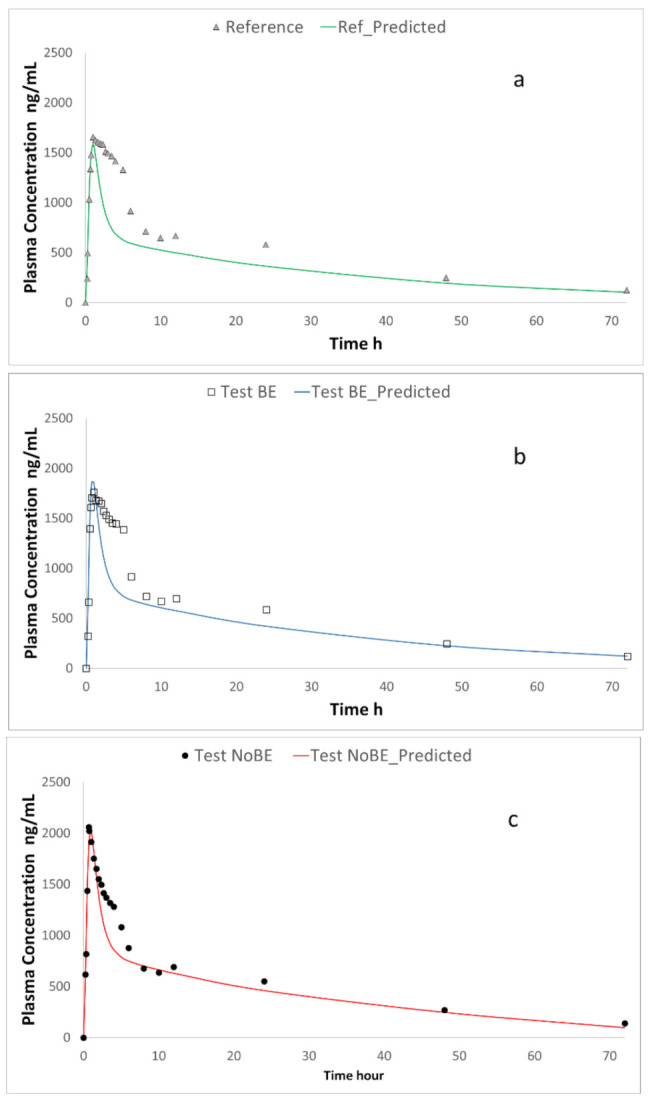
Experimental and predicted plasma profiles for the three studied formulations Plot (**a**), Reference formulation; Plot (**b**), Bioequivalent (BE) formulation; Plot (**c**), Non BE formulation.

Plasma profiles of ETO products were simulated by a two-compartmental in silico PK model. The in vitro dissolution profiles from each ETO product in duodenal and jejunal chambers were used as the input function to be convoluted with the disposition parameters obtained from literature. The predicted outcome is illustrated in Figure 4.

Plasma concentration profiles of the three products were predicted and the C_max_ and AUC predicted values were calculated by non-compartmental methods. In summary, the parameters from Table 3 were convoluted with the PK parameters in Table 4 in WinNonlin software using the equations described in the appendix to get the in vivo predicted plasma levels. Table 5 shows the prediction errors of C_max_ values. Table 6 and Table 7 summarizes the experimental and predicted C_max_ ratios and AUC data, respectively.

## 4. Discussion

The GIS was able to detect the impact of excipients on the release and dissolution of the ETO in the stomach, as well as its precipitation in duodenum and jejunum, which caused different concentrations at the absorptive sites and, consequently, different systemic peak exposure. The in vitro dissolution results along with in silico simulations indicated that one generic product candidate would be bioequivalent with the reference product, whereas the other generic product candidate would not be bioequivalent. These predictions agreed with the systemic exposure data obtained from cross-over BE studies.

ETO, as a weakly basic compound, has higher solubility in the acidic environment of the stomach (pH 2) and could induce supersaturation and precipitation after gastric emptying. Despite the high solubility in the stomach (13.21 mg/mL in pH 1.2 buffer according to Okumu et al. [7] and 25 mg/mL according to Mitra el al. [6]), dissolution was not complete in the stomach and (undissolved) drug particles were transferred to the duodenal chambers where, a maximum concentration around 0.35 mg/mL was observed, which is far from its thermodynamic solubility. This incomplete dissolution in the stomach can be explained as the dissolution rate becomes relatively slow because the acidic content is neutralized by the dissolving free bases at the solid surface [22], i.e., the surface pH of drug particles becomes higher than the bulk pH, dictating a lower solubility value at the solid surface and thus slowing down dissolution rate. The difference among the three oral tablets becomes evident already in gastric chamber. A potential explanation might be the presence of calcium phosphate in the reference product, which attributes the lower concentration and amount dissolved. We have shown recently that in formulations of dexketoprofen trometamol (salt of a weak acid), excipients that increase pH (calcium phosphate) decreased free acid precipitation and enhanced dissolved levels of drug [20]. In the case of the weak base the same effect would lead to a slower dissolution rate due to the higher pH around the solid particles. Actually, Schwartz et al. [23] showed that ETO plasma C_max_ values were reduced by 23% and 15% by calcium carbonate, magnesium and aluminum hydroxide, respectively.

Apart from the negative effect of calcium phosphate on the dissolution of ETO present in the reference product, a faster disintegration was visually observed in GIS conditions for both generic products, probably due to a larger amount of sodium croscarmellose, which could also justify the faster dissolution and the higher and sustained supersaturation in jejunal conditions. The fitted dissolution coefficient Z was higher for the test products.

All drug products contained sodium croscarmellose. BE and non-BE products contain sodium croscarmellose, but a reduced amount of croscarmellose was observed in the BE product that was compensated with microcrystalline cellulose to obtain the same core weight in both generic formulations. Sodium croscarmellose, as other cellulose-based polymers, has shown to act as precipitation inhibitor [24]. The different content of this disintegrant and microcrystalline cellulose in BE versus non-BE product (2.05% with respect to the core weight, which is close to the 2% limit defined for disintegrants other than starch for granting a BCS biowaiver for class III drugs in the ICH M9 guideline) could be the reason for the higher supersaturation level observed in the NoBE product. This effect is reflected in the lower precipitation rate coefficient k_pre_ 3.2 × 10^−2^ h^−1^ for the non-BE vs. 8.3 × 10^−2^ h^−1^ in the BE. 

The dissolved amounts in the duodenal chamber maintained the same rank order as generated in the stomach. Okumu et al. [7] did not observe precipitation in their transfer system. They explained this fact based of the relatively low supersaturation ratio (i.e., calculated as the ratio of dissolved concentration divided by the equilibrium solubility), which was below 3. In the GIS device, the maximum observed ETO concentrations in duodenum reached 0.3 mg/mL versus the reported solubility at pH 4.5 of 0.44 mg/mL. A low supersaturation ratio will result in a minimal chance of precipitation as not that much drug molecules are in a supersaturated state, finding each other to precipitate. In the jejunal chamber, sustained supersaturation concentrations were observed for all the products (final concentrations higher than 0.2 mg/mL (ETO solubility of 0.14 mg/mL in FaSSIF or simulated intestinal fluids at pH 6.8) in agreement with Okumu et al. observations, but the concentration of both test products were higher compared to the reference.

Although we did not use bile salts and lecithin to form colloidal structures that could benefit the solubilization of the compound, we believe that these constituents will not have a major impact on supersaturation, precipitation and solubilization as the reported solubility values in FaSSIF and SIF phosphate buffer are both the same (i.e., 0.14 mg/mL) [7]. ETO has a LogP of 2.794, and according to Mudie et al. [25] the presence of bile salts is not expected to affect dissolution rate when logP < 3.

The predicted plasma profiles, convoluted from the in vitro data, reproduced correctly the rank order of the plasma C_max_ values, and the prediction errors of C_max_ values were below 10%. AUC values were underpredicted for all the products mainly due to the underpredicted plasma values between 5 and 10 h. The difference between (i) the actual disposition parameters as observed from the BE studies and (ii) the parameters from Mitra et al. [6] could be the reason. Another explanation is the observed secondary peak (previously reported by other authors [23], whose origin is not clear [8]), which could be due to the enterohepatic cycling of the glucuronide metabolite [10], however, this process was not included in the PK model. On the other hand, the predicted AUC ratios are similar for both products as observed in vivo. The predictions showed the same rank order than the experimental ratios. Predictions were obtained with a deterministic model not including parameter variability or residual variability so we do not have a confidence interval for the predictions. Nevertheless, if used in the developed setting to inform about the inequivalence risk, the proposed dissolution method would have identified the “non-BE” formulation as the one with higher risk of not passing the BE standards

There are two previous studies using a computational model combined with in vitro dissolution to predict plasma ETO levels. Okumu et al. [7], established an IVIVR for the IR tablet *Arcoxia*^®^. using the Gastroplus™ simulator. They concluded that 0.01 M HCl and FaSSIF media (both in USP II at 50 rpm) were the best conditions for predicting in vivo systemic concentrations. Mitra el al. [6] used a similar mechanistic absorption model and validated the model based on clinical data where subjects received different doses of ETO under fasted and achlorhydric conditions (i.e., elevated gastric pH levels due to concomitant antacid administration). The in vitro profiles used as input in Gastroplus™ were obtained in USP II at 50 rpm in different media at pH 2 (0.01 M HCl), pH 4.5 (acetate buffer) and pH 6.8 (phosphate buffer). While this approach adds information from different batches, all of them correspond to the same formulation, thus the potential influence of excipients cannot be distinguished/differentiated in the generated dissolution profiles and, consequently, in the predicted plasma levels. Consequently, this study presents for the first time the in vitro-in vivo correlation among formulations with different in vivo dissolution rate (evidenced in the BE study outcomes).

Mitra et al. concluded that absorption rate and extent of ETO will be determined by the initial dissolution in the stomach environment (pH < 3) as confirmed by Okumu et al. [7] and in accordance with our present results. Okumu et al. also pointed out the potential relevance of the dissolution in FaSSIF, while Mitra and co-workers reported that differences in pH 4.5 and 6.8 were not reflected in differences in in vivo systemic outcomes. In our study, the products showed similarity in the BCS-biowaiver media (pH 1.2 at 50 rpm, and 4.5 and 6.8 at 75 rpm to avoid coning effect in USP II, Figure S1 in Supplementary Material), thus the predictability of the BCS media in USP II apparatus for different products was not adequate, while the dissolution profiles obtained in the GIS coupled with the in silico model were predictive with respect to the BE outcome.

## 5. Conclusions

In the present work, the GIS pointed out the impact of excipients on the release and dissolution of the ETO in the stomach, as well as its precipitation in duodenum and jejunum, which led to different concentrations at the absorptive sites. The in vitro dissolution results with in silico simulations indicated that one generic product candidate would be bioequivalent with the reference product, whereas the other generic product candidate would not be bioequivalent. These predictions agreed with the systemic exposure data obtained from crossover BE studies. The difference observed in the dynamic setup of the GIS could not be observed in the traditional USP II apparatus, suggesting the importance of multistage dissolution testing for weak-base drugs to capture the interplay of dissolution, precipitation and transit times under physiological pH changes. Absorption modelling combined with physiologically relevant dissolution profiles has shown to be a valuable tool to be used in formulation development and could support in the future, when more experience is gained, the waiver of in vivo BE studies instead of performing labor-intensive, time consuming, and expensive in vivo BE studies.

## Figures and Tables

**Table 1:** Ratio Test/Reference of plasma maximal concentration, C_max_ and area under the curve AUC_0-72h_ values and 90 confidence intervals in both human bioequivalence studies BE.

Study	Study 1 Failed to Conclude BE		Study 2 BE Concluded	
Parameter	Ratio Test/Reference	90% Confidence Interval	Ratio Test/Reference	90% Confidence Interval
C_max_	118.20	111.26–125.57	112.57	104.27–121.54
AUC_0–72h_	100.48	97.54–103.50	102.96	99.10–106.97

**Table 2:** Experimental conditions in the Gastrointestinal Simulator, GIS for testing the different drug products of Etoricoxib.

Fasted State Test Conditions	GIS_Stomach_	GIS_Duodenum_	GIS_Jejunum_
Dissolution Media	Simulated Gastric Fluid (SGF), pH 2.0, 0.01 M HCl + 34.2 mM NaCL	Phosphate Buffer 5 mM pH 6.5	/
Initial Volume	50 mL SGF + 250 mL of water	50 mL	/
Secretions	1 mL/min of SGF	1 mL/min of Phosphate Buffer 100 mM pH 6.5	/

**Table 3:** Input parameters and fitted ones for describing the dissolution, precipitation and transit kinetics of ETO for three different oral products in the GIS. k_sec_s_ and k_sec_d_: secretion rates in stomach and duodenum respectively; t_1/2G_: gastric emptying half-life; V_s_, V_d_ and V_j_: volumes in stomach, duodenum and jejunum; Z: dissolution rate coefficient; kpre: precipitation rate constant; Cs: solubility values at each pH.

Parameter	ETO NoBE	ETO BE	Reference Product	Reference
Dose (mg)	120	120	120	
k_sec_s_ (mL/min)	1	1	1	[20]
k_sec_d_ (mL/min)	1	1	1	[20]
t_1/2,G_ (min)	13	13	13	[20]
V_s_ (mL)	300 to 5	300 to 5	300 to 5	[20]
V_d_ (mL)	50	50	50	[20]
V_j_ (mL)	0 to 390	0 to 390	0 to 390	[20]
Z (mL/mg/min)	3.51 × 10^−5^	3.10 × 10^−5^	1.45 × 10^−5^	Optimized by fitting
kpre (min^−1^)	3.18 × 10^−2^	8.30 × 10^−2^	7.84 × 10^−2^	Optimized by fitting
Cs mg/mL pH 2.0	13.21	13.21	13.21	[6,7]
Cs mg/mL pH 4.5	0.44	0.44	0.44	[7]
Cs mg/mL pH 6.8	0.14	0.14	0.14	[7]

ksec_s and ksec_d respectively represent the secretion rates in the gastric and duodenal chamber. t_1/2,G_ stands for the gastric half-life of emptying; Volumes are denoted as Vs, Vd, and Vj for the gastric, duodenal, and jejunal chambers. Z is the dissolution coefficient. Precipitation is described as first-order kinetic process with precipitation rate coefficient kpre.

**Table 4:** Systemic PK parameters obtained from curve fitting of literature data applied for in silico modeling of ETO in order to predict the plasma levels from in vitro dissolution profiles.

Pharmacokinetic (PK) Parameters	Value
Central compartment volume—Vc (L) Average value from [6,9,21]	27.40
Elimination rate constant from central compartment—ke (h^−1^) [6]	0.0899
Distribution rate constant from central to peripheral compartment—k12 (h^−1^) [6]	0.6180
Distribution rate constant from peripheral to central compartment—k21 (h^−1^) [6]	0.2820
Effective Permeability Small Intestine—Peff (cm/h) [6]	1.71

**Table 5:** Plasma C_max_ prediction errors for the three studied products. PE: prediction error

Parameter	Reference	BE	NoBE
C_max_ experimental ng/mL	1657	1762	2060
C_max_ predicted ng/mL	1583	1866	2017
PE %	4.4	−5.9	2.1

**Table 6:** Predicted and experimental ratios (Test/Reference) of plasma C_max_ for both products.

C_max_ Ratio	Ratio Predicted	Ratio Experimental
Test BE	1.18	1.12
Test NoBE	1.27	1.18

**Table 7:** Plasma area under the curve (AUC) predicted and experimental values and predicted and experimental ratios.

AUC_(0–72)_ ng/mL × h	Reference	BE	NoBE
Experimental	38,101	38,693	38,749
Predicted	28,009	32,303	32,854
Ratio exp.		1.02	1.02
Ratio pred.		1.15	1.17

## Data Availability

The raw in vitro dissolution data presented in this study are available on request from the corresponding author. The data from the clinical study, apart from the average data presented here, are not publicly available due to confidentiality reasons.

## Supplementary Materials

The following are available online at https://www.mdpi.com/article/10.3390/pharmaceutics13040507/s1. Figure S1: Dissolution profiles of Reference (solid circles) and Test Formulations (solid triangles) of ETO in USP II apparatus at pH 1.2, 4.5 and 6.8.
