# Supplementary material for: An In Vivo Predictive Dissolution Methodology (iPD Methodology) with a BCS Class IIb Drug Can Predict the In Vivo Bioequivalence Results: Etoricoxib Products
Source: Pharmaceutics. 2021 Apr 7;13(4):507. doi: 10.3390/pharmaceutics13040507 (PMC8067797; doi:10.3390/pharmaceutics13040507)
Supplement: Supplementary file 1 [file pharmaceutics-13-00507-s001.pdf]

pharmaceutics

MDPI

Supplementary Materials: An In Vivo Predictive Dissolution Methodology (iPD Methodology) with a BCS Class IIb Drug Can Predict the In Vivo Bioequivalence Results: Etoricoxib Products

Isabel Gonzalez-Alvarez, Marival Bermejo, Yasuhiro Tsume, Alejandro Ruiz-Picazo, Marta Gonzalez-Alvarez, Bart Hens, Alfredo Garcia-Arieta, Greg E. Amidon and Gordon L. Amidon

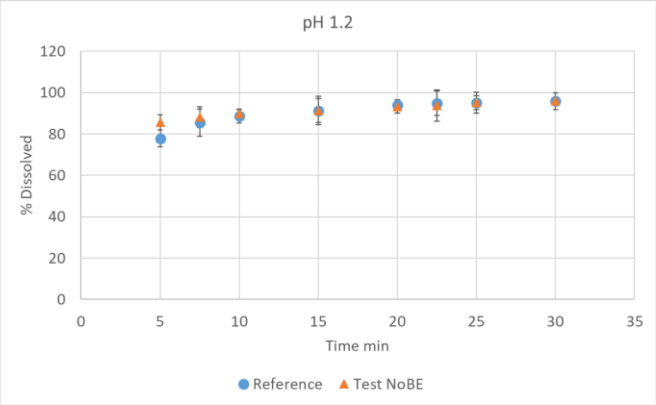

a) F2 = N.A

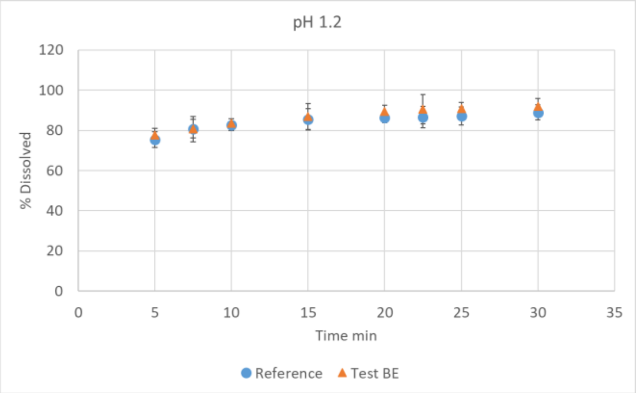

b) F2 = N.A

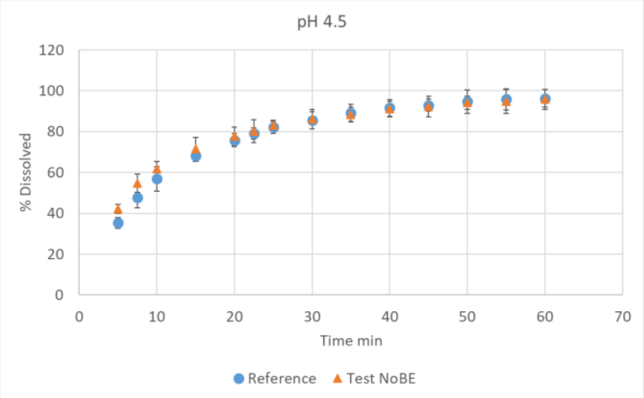

c) F2 = 68.3

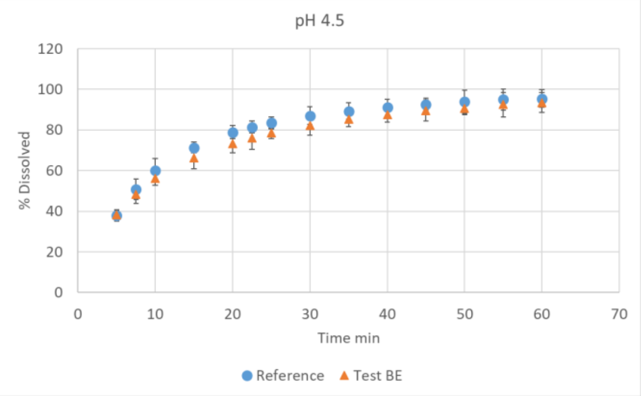

d) F2 = 66.94

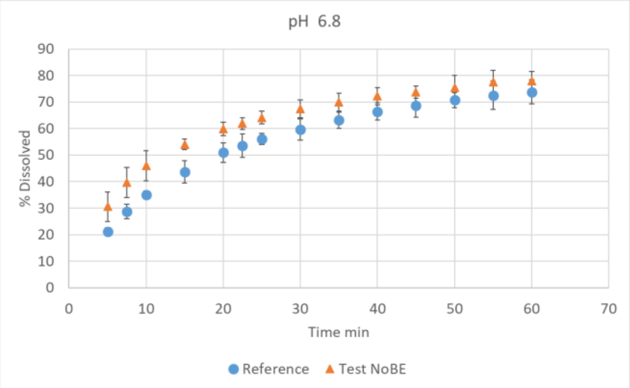

e) F2 = 54.90

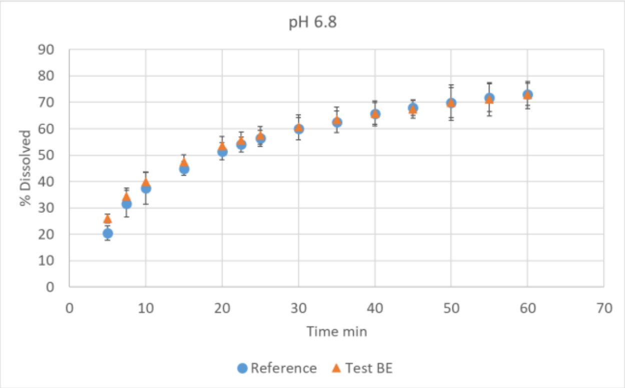

f) F2 = 82.36

Figure S1. Dissolution profiles of Reference (solid circles) and Test Formulations (solid triangles) of ETO in USP II apparatus at pH 1.2, 4.5 and 6.8. Values are the average of six tablets. Similarity factor F2 is indicated when its calculation was possible. N.A: not applicable. (**a**) Comparison Reference *versus* Test NoBE at pH 1.2; (**b**) Comparison Reference *versus* Test BE at pH 1.2; (**c**) Comparison Reference *versus* Test NoBE at pH 4.5; (**d**) Comparison Reference *versus* Test BE at pH 4.5; (**e**) Comparison Reference *versus* Test NoBE at pH 6.8; (**f**) Comparison Reference *versus* Test BE at pH 6.8.

Definition of terms:

$X_{ud(segment)}$ = undissolved amount in each GIS chamber or intestinal segment
$X_{d(segment)}$ = dissolved amount in each GIS chamber or intestinal segment
X_{cen} = amount in central compartment
X_{peri} = amount in peripheral compartment
$V_{segment}$ = volume in each GIS chamber or intestinal segment
$V_{segment,0}$ = initial volume in each GIS chamber or intestinal segment
$k_{sec(segment)}$ = *s*ecretion rate constant in each GIS chamber or intestinal segment
$C_{end(segment)}$ = final concentration in each GIS chamber or intestinal segment
Segment = (stomach (s), duodenum(d), jejunum(j))
F_{sys} = systemic availability; 1
R = intestinal radious; 1.5 cm
DF = degree of flatness; 2
kt = transit constant from jejunum to distal segments: 0.0056 min^{-1} [1]
Parameters Z (dissolution parameters) and kpre (precipitation parameter)

Mass Equations for GIS

Equation (S1): Undissolved drug in the $GIS_{stomach}$:

$$\frac{dX_{ud(s)}}{dt} = -Z \cdot (X_{d(s)} + X_{ud(s)})^{1/3} {X_{ud(s)}}^{2/3} \left(C_{s(s)} - \frac{X_{d(s)}}{V_s}\right) - \left(-\frac{dV_s}{dt} + k_{sec(s)}\right) \left(\frac{X_{ud(s)}}{V_s}\right) \tag{S1}$$

Equation (S2): Dissolved drug in the $GIS_{stomach}$:

$$\frac{dX_{d(s)}}{dt} = Z \cdot (X_{d(s)} + X_{ud(s)})^{1/3} {X_{ud(s)}}^{2/3} \left(C_{s(s)} - \frac{X_{d(s)}}{V_s}\right) - \left(-\frac{dV_s}{dt} + k_{sec(s)}\right) \left(\frac{X_{d(s)}}{V_s}\right) \tag{S2}$$

Equation (S3): Undissolved drug in the $GIS_{duodenum}$:

$$\frac{dX_{ud(d)}}{dt} = -Z \cdot \left(X_{d(d)} + X_{ud(d)}\right)^{1/3} {X_{ud(d)}}^{2/3} \left(C_{s(d)} - \frac{X_{d(d)}}{V_d}\right) + k_{pre(d)}\left(X_{d(d)} - C_{end(d)} V_d\right) + \left(-\frac{dV_s}{dt} + k_{sec(s)}\right)\left(\frac{X_{ud(s)}}{V_s}\right) - \left(-\frac{dV_s}{dt} + k_{sec(s)} + k_{sec(d)}\right)\left(\frac{X_{ud(d)}}{V_d}\right) \tag{S3}$$

Equation (S5): Dissolved drug in the $GIS_{duodenum}$:

$$\frac{dX_{d(d)}}{dt} = Z \cdot \left(X_{d(d)} + X_{ud(d)}\right)^{1/3} {X_{ud(d)}}^{2/3} \left(C_{s(d)} - \frac{X_{d(d)}}{V_d}\right) - k_{pre(d)}\left(X_{d(d)} - C_{end(d)} V_d\right) + \left(-\frac{dV_s}{dt} + k_{sec(s)}\right)\left(\frac{X_{d(s)}}{V_s}\right) - \left(-\frac{dV_s}{dt} + k_{sec(s)} + k_{sec\,(d)}\right)\left(\frac{X_{d(d)}}{V_d}\right) \tag{S4}$$

Equation (S5): Undissolved drug in the $GIS_{jejunum}$:

$$\frac{dX_{ud(j)}}{dt} = -Z\,(X_{d(j)} + X_{ud(j)})^{1/3} {X_{ud(j)}}^{2/3}\left(C_{s(j)} - \frac{X_{d(j)}}{V_j}\right) + k_{pre(j)}\big(X_{d(j)} - C_{end(j)}V_j\big) + \left(-\frac{dV_s}{dt} + k_{sec(s)}\right)\left(\frac{X_{d(s)}}{V_s}\right) - \left(-\frac{dV_s}{dt} + k_{sec(s)} + k_{sec\,(d)}\right)\left(\frac{X_{d(d)}}{V_d}\right) \quad (S5)$$

Equation (S6): Dissolved drug in the $GIS_{jejunum}$:

$$\frac{dX_{d(j)}}{dt} = Z \cdot (X_{d(j)} + X_{ud(j)})^{1/3} {X_{ud(j)}}^{2/3}\left(C_{s(j)} - \frac{X_{d(j)}}{V_j}\right) - k_{pre(j)}(X_{d(j)} - C_{end(j)}V_j) + \left(-\frac{dV_s}{dt} + k_{sec(s)} + k_{sec\,(d)}\right)\left(\frac{X_{d(d)}}{V_d}\right) \quad (S6)$$

Equation (S7): Volume in stomach:

$$V_s = V_{s,0} \times e^{-\left(\frac{\ln(2)}{GE}\right)t} \quad [mL] \quad (S7)$$

(if $t > 60$ min, $t = 60$)

Equation (S8): Volume in duodenum:

$$V_d = V_{d,0} \quad [mL] \quad (S8)$$

Equation (S9): Volume in jejunum:

$$V_j = V_{s,0} \times \left(1 - e^{-\left(\frac{\ln(2)}{GE}\right)t}\right) + \big(k_{sec(s)} + k_{sec(d)}\big) \times t \quad [mL] \quad (S9)$$

(if $t > 60$ min, $t = 60$)

Equation (S10): secretion rate in stomach:

$$k_{sec(s)} = 0.001 \left[\frac{L}{min}\right] \quad (S10)$$

(if $t > 60$ min, $k_{sec\,(s)} = 0$)

Equation (S11): secretion rate in duodenum:

$$k_{sec(d)} = 0.001 \left[\frac{L}{min}\right] \quad (S11)$$

(if $t > 60$ min, $k_{sec\,(d)} = 0$)

Mass Equations for in vivo plasma predictions

Equation (S12): Undissolved drug in the stomach:

$$\frac{dX_{ud(s)}}{dt} = -Z \cdot (X_{d(s)} + X_{ud(s)})^{1/3} {X_{ud(s)}}^{2/3}\left(C_{s(s)} - \frac{X_{d(s)}}{V_s}\right) - \left(-\frac{dV_s}{dt} + k_{sec(s)}\right)\left(\frac{X_{ud(s)}}{V_s}\right) \quad (S12)$$

Equation (S13): Dissolved drug in the stomach:

$$\frac{dX_{d(s)}}{dt} = z_{(s)}\big(X_{d(s)} + X_{ud(s)}\big)^{1/3} {X_{ud(s)}}^{2/3}\left(C_{s(s)} - \frac{X_{d(s)}}{V_s}\right) - \left(-\frac{dV_s}{dt} + k_{sec(s)}\right)\left(\frac{X_{d(s)}}{V_s}\right) \quad (S13)$$

Equation (S14): Undissolved drug in the duodenum:

$$\frac{dX_{ud(d)}}{dt} = -Z \cdot \big(X_{d(d)} + X_{ud(d)}\big)^{1/3} {X_{ud(d)}}^{2/3}\left(C_{s(d)} - \frac{X_{d(d)}}{V_d}\right) + k_{pre(d)}\big(X_{d(d)} - C_{end(d)}V_d\big) + \left(-\frac{dV_s}{dt} + k_{sec(s)}\right)\left(\frac{X_{ud(s)}}{V_s}\right) - \left(-\frac{dV_s}{dt} + k_{sec(s)} + k_{sec(d)}\right)\left(\frac{X_{ud(d)}}{V_d}\right) \quad (S14)$$

Equation (S15): Dissolved drug in the duodenum:

$$\frac{dX_{d(d)}}{dt} = Z \cdot (X_{d(d)} + X_{ud(d)})^{1/3} X_{ud(d)}{}^{2/3} \left(C_{s(d)} - \frac{X_{d(d)}}{V_d}\right) - k_{pre(d)}\left(X_{d(d)} - C_{end(d)}V_d\right) + \left(-\frac{dV_s}{dt} + k_{sec(s)}\right)\left(\frac{X_{d(s)}}{V_s}\right) - \left(-\frac{dV_s}{dt} + k_{sec(s)} + k_{sec(d)}\right)\left(\frac{X_{d(d)}}{V_d}\right) - \frac{2DF}{R} X_{d(d)} P_{eff} \quad \text{(S15)}$$

Equation (S16): Undissolved drug in the jejunum:

$$\frac{dX_{ud(i)}}{dt} = -Z \cdot \left(X_{d(i)} + X_{ud(i)}\right)^{1/3} X_{ud(i)}{}^{2/3} \left(C_{s(i)} - \frac{X_{d(i)}}{V_i}\right) + k_{pre(i)}\left(X_{d(i)} - C_{end(i)}V_i\right) + \left(-\frac{dV_s}{dt} + k_{sec(s)} + k_{sec(d)}\right)\left(\frac{X_{ud(d)}}{V_d}\right) - k_t X_{ud(i)} \quad \text{(S16)}$$

Equation (S17): Dissolved drug in the jejunum

$$\frac{dX_{d(i)}}{dt} = Z \cdot \left(X_{d(i)} + X_{ud(i)}\right)^{1/3} X_{ud(i)}{}^{2/3} \left(C_{s(i)} - \frac{X_{d(i)}}{V_i}\right) - k_{pre(i)}\left(X_{d(i)} - C_{end(i)}V_i\right) + \left(-\frac{dV_s}{dt} + k_{sec(s)} + k_{sec(d)}\right)\left(\frac{X_{d(d)}}{V_d}\right) - k_t X_{d(i)} - \frac{2DF}{R} X_{d(i)} P_{eff} \quad \text{(S17)}$$

Equation (S18): Drug in the central compartment:

$$\frac{dX_{cen}}{dt} = \left(\frac{2DF}{R} X_{d(d)} P_{eff} + \frac{2DF}{R} X_{d(i)} P_{eff}\right) F_{Sys} - k_{12} X_{cen} - k_{13} X_{cen} + k_{21} X_{peri1} + k_{31} X_{peri2} - k_e X_{cen} \quad \text{(S18)}$$

Equation (S19): Drug in the peripheral compartment 1:

$$\frac{dX_{peri1}}{dt} = k_{12} X_{cen} - k_{21} X_{peri1} \quad \text{(S19)}$$

Equation (S20): Drug in the peripheral compartment 2:

$$\frac{dX_{peri2}}{dt} = k_{13} X_{cen} - k_{31} X_{peri2} \quad \text{(S10)}$$

Equation (S21): Plasma drug concentration:

$$C_p = \frac{X_{cen}}{V_c} \quad \text{(S11)}$$

References:

1 K. Matsui, Y. Tsume, S. Takeuchi, A. Searls, G.L. Amidon, Utilization of Gastrointestinal Simulator, an in Vivo Predictive Dissolution Methodology, Coupled with Computational Approach To Forecast Oral Absorption of Dipyridamole, *Mol. Pharm.* **2017**, *14*, 1181–1189, doi.org/10.1021/acs.molpharmaceut.6b01063.
